# Acoustic Oddball during NREM Sleep: A Combined EEG/fMRI Study

**DOI:** 10.1371/journal.pone.0006749

**Published:** 2009-08-25

**Authors:** Michael Czisch, Renate Wehrle, Andrea Stiegler, Henning Peters, Katia Andrade, Florian Holsboer, Philipp G. Sämann

**Affiliations:** Max Planck Institute of Psychiatry, Munich, Germany; Ludwig Maximilians University Munich, Germany

## Abstract

**Background:**

A condition vital for the consolidation and maintenance of sleep is generally reduced responsiveness to external stimuli. Despite this, the sleeper maintains a level of stimulus processing that allows to respond to potentially dangerous environmental signals. The mechanisms that subserve these contradictory functions are only incompletely understood.

**Methodology/Principal Findings:**

Using combined EEG/fMRI we investigated the neural substrate of sleep protection by applying an acoustic oddball paradigm during light NREM sleep. Further, we studied the role of evoked K-complexes (KCs), an electroencephalographic hallmark of NREM sleep with a still unknown role for sleep protection. Our main results were: (1) Other than in wakefulness, rare tones did not induce a blood oxygenation level dependent (BOLD) signal increase in the auditory pathway but a strong negative BOLD response in motor areas and the amygdala. (2) Stratification of rare tones by the presence of evoked KCs detected activation of the auditory cortex, hippocampus, superior and middle frontal gyri and posterior cingulate only for rare tones followed by a KC. (3) The typical high frontocentral EEG deflections of KCs were not paralleled by a BOLD equivalent.

**Conclusions/Significance:**

We observed that rare tones lead to transient disengagement of motor and amygdala responses during light NREM sleep. We interpret this as a sleep protective mechanism to delimit motor responses and to reduce the sensitivity of the amygdala towards further incoming stimuli. Evoked KCs are suggested to originate from a brain state with relatively increased stimulus processing, revealing an activity pattern resembling novelty processing as previously reported during wakefulness. The KC itself is not reflected by increased metabolic demand in BOLD based imaging, arguing that evoked KCs result from increased neural synchronicity without altered metabolic demand.

## Introduction

Sleep, as compared to wakefulness, is characterized by altered and reduced reactivity to external stimulation, but the brain mechanisms underlying these fundamental changes are not yet fully disclosed. On the other hand, some stimulus processing must be preserved allowing the sleeper to be aroused by personally meaningful or threatening stimuli. So far, mostly electrophysiological techniques, especially averaged event-related potentials (ERPs), have been used to investigate the brain's responsiveness to external stimuli, since electrophysiological measures are independent of behavioral responses or conscious awareness. In NREM sleep, several large amplitude ERP components are thought to reflect inhibition of information processing and may reflect the brain's capacity to generate delta frequency EEG activity, a marker of deep slow wave sleep [1].

The oddball paradigm, being a simple discrimination task, has been widely used to probe attentiveness in different vigilance states [1]–[5]. While active detection of stimulus deviance is associated with a P300 component during wakefulness, this component is not detectable when subjects either ignore or fail to detect the rare tone, or in consolidated NREM sleep [1], [5]. Similar, a mismatch negativity reflecting pre-attentive stimulus discrimination processes can be recorded during wakefulness, but is strongly reduced or absent in light NREM sleep [6], [7]. Furthermore, during NREM sleep an attenuation of the early N1 component has been observed, while P2 is enhanced, hypothesized to reflect decreased cortical excitability and increased inhibitory processes [1], [5], [8], [9]. In addition, sleep specific late ERP components such as the N350, P450, N550 and P900 arise. These components are most enhanced subsequent to rare stimuli, and have been found strongest with increased sleep pressure during the first half of the night [4] or following sleep fragmentation or sleep deprivation [10]–[12]. Taken together, it has been suggested that these late ERP components reflect inhibitory processes minimizing cognitive processing.

The late components N550 and P900 in NREM sleep are a reflection of the so called K-complex (KC), first described by Loomis et al. [13] as a prominent element of the human sleep EEG (for recent reviews, see e. g. [14], [15]). KCs may appear spontaneously, but can also be evoked by external stimuli. The functional significance of KCs, however, is still a matter of debate. As KCs can be elicited by cortical, thalamic or sensory stimuli, they are obviously involved in some form of information processing during NREM sleep. This has led to the notion of KCs being reactive micro-arousal processes without awakening [16]. Compatible with this notion, cortical reactivity was found increased in periods preceding KCs [17]. In turn, it has been hypothesized that KCs may also serve as a sleep protective mechanism by triggering anti-arousal reactions that support the consolidation and maintenance of sleep [15], [18]–[20]. Furthermore, Amzica and Steriade [21] suggested that KCs may represent the alternation between a state in which the cortical network would be ready to operate in case of danger (depolarizing phase) and a state where the brain is at rest, allowing replenishing of cellular energetic stores (hyperpolarizing phase).

First fMRI studies performed during NREM sleep applying acoustic stimulation showed contradictory results: Portas et al. [22] as well as Redcay et al. [23] and Wilke et al. [24] identified preserved activation of the primary auditory cortex in light NREM sleep in adults and children, respectively. However, these studies did not include EEG measurements to validate sleep during the actual fMRI data acquisition. The use of simultaneous EEG recordings and analysis of epochs directly related to sound presentation is mandatory to objectify sleep stages and to affirm sleep continuity. Other groups showed reduced or even inverted BOLD responses in the auditory cortex [25]–[27] in electrophysiologically verified sleep stages 1 and 2, respectively. All studies used different acoustic stimuli, e.g. personally relevant stimuli with strong arousal capacity such as the subject's own name, or less arousing stimuli like classical music or pure tones. In our earlier studies we observed a negative BOLD response (NBR) to acoustic stimuli presented in a block design during light NREM sleep, with paralleled increases in occurrence of KCs and delta activity [26], [27]. Using a different sensory modality, an NBR has also been obtained by Born et al. [28] for the primary and secondary visual cortex in response to appropriate visual stimuli during early NREM sleep. We proposed this NBR to reflect sleep inhibitory processes that eventually renders a function in consolidation and deepening of sleep.

Extending previous findings utilizing a block-presentation of stimulation, the present study aimed at a more precise description of auditory processing during light NREM sleep stage 2. To do so, an event-related acoustic oddball paradigm with rare tones interspersed in more frequent tones was applied. Experiments were designed to test the following hypotheses:

1. Response to the oddball paradigm during sleep is altered as compared to wakefulness. Especially, we hypothesize a reduced or even inverted BOLD response in the auditory cortex.
2. Based on our previous findings, a general sleep defensive mechanism is postulated to be triggered by rare tones which should be reflected by an crossmodal NBR also in brain areas not directly targeted by the acoustic paradigm.
3. The oddball paradigm has been shown to evoke KCs during sleep stage 2. By separating rare tone segments with or without KCs we aimed at identifying the neuronal representation of evoked KCs in BOLD based functional imaging. KC related cerebral activity is predicted mainly to be located in frontal and central brain areas, as evidenced by the frontocentral maximum of KCs in surface EEG recordings.

## Materials and Methods

### Ethics Statement

The study protocol followed the guidelines of the Declaration of Helsinki and was approved by the local ethical committee (Bayrische Landesärztekammer, Germany, Nr. 01102).

### Subjects

Young healthy subjects were recruited by public advertising and they gave written informed consent prior to the study. All participants underwent a careful screening including a structured clinical interview, a medical examination, routine blood tests and drug screening, clinical MRI and EEG including polysomnographic recordings. Exclusion criteria were any lifetime axis I psychiatric diagnosis according to a computerized DSM-IV version of the Munich-Composite International Diagnostic Interview [29], sleep-related disorders or circadian rhythms exceeding intermediate or moderate morning- or evening-types [30], consumption of drugs, consumption of more than 2 cups of coffee per day or more than 5 alcoholic drinks per week, any regular medication, crossing of time zones during the 3 months before the study and any contraindications to MRI. All subjects were right-handed and non-smokers.

In total, 18 volunteers (9 m/9 f, mean age 25.4±2.5 years) underwent the study protocol. Subjects had to follow a regular sleep-wake-schedule for one week before the experiments. The first experimental session was recorded during wakefulness (starting at about 17:00 hrs, duration: 3′40″ minutes), and the second session was recorded while subjects were trying to fall asleep in the MRI scanner (start at about 21:00 hrs, 26′40″ minutes). To increase sleep pressure, subjects were asked to get up three hours earlier than usually. If the subject was unable to fall asleep during the first run as visible in the online simultaneous EEG, the sleep experiment was immediately repeated a second time.

### Acoustic stimulation

For acoustic stimulation we used a passive two tone oddball paradigm programmed using the Presentations Software (Neurobehavioral Systems, Albany, USA). The paradigm has been chosen because it has been frequently described in ERP studies to assess sleep specific responses to frequent and rare tones. Furthermore, it was favored over simple presentations of rare tones only due to a more homogenous acoustic background created by the frequent tones, supposed to have less adverse effects on sleep continuity. In our paradigm, rare *odd* tones (1500 Hz, duration 50 ms) appeared with 20% probability against the background of frequent tones (1000 Hz, duration 50 ms). The interstimulus interval was set to 2000 ms as this period allows to catch potentially elicited KC events in the ERP data, and assigned with a random jitter of±0, ±100 or ±200 ms. The order of the tones was randomly assigned. Still, two consecutive rare tones were always separated by at least two frequent tones and the 20% rare tone probability had to be fulfilled in subsets of 20 tones. Acoustic stimuli were delivered by a magnetostatic headphone (MR-Confon, Magdeburg, Germany). In addition, subjects wore foam ear plugs for safety reasons. To adjust loudness of the acoustic stimuli, a preparation scan was performed during which subjects had to repeatedly decide whether or not they perceived the tones as loud as the fMRI scanner sound. This resulted in a defined level of subjectively identical loudness of tones and scanner noise. For the final experiment, tones were delivered 3 dB louder for better perception (within an absolute range of 80–85 dB). The main acoustic frequency component of the fMRI sequence was about 800 Hz, outside the acoustic spectrum of frequent and rare tones. After the loudness adjustment, subjects were instructed to listen to the tones without focussing on different tone types. We also explained that no active response is requested. Last, in the sleep condition, subjects were told that falling asleep was part of the experiment and should not be actively prevented.

### FMRI acquisition

Whole brain functional images were acquired on a 1.5T GE Signa Excite System (Milwaukee, USA) using an 8-channel phased array head coil. 25 slices (64×64 points, 3 mm thickness, 1 mm gap, AC-PC orientation) were recorded per EPI volume (TR 2 s, TE 40 ms). In total, 800 fMRI volumes were measured while the subject was falling asleep during oddball presentation. For comparison, independent data were recorded in a separate session during wakefulness by applying the same acoustic paradigm without requesting a response (passive oddball). Here, two consecutive fMRI experiments of 3′40″ minutes each were collected in all n = 18 subjects. All fMRI data were collected with simultaneous EEG recordings.

### Polysomnographic recording

For simultaneous polysomnographic recording, special hardware compatible with the magnetic field was used (MRplus 32-channels, Brain Products, Gilching, Germany). 19 EEG surface electrodes were placed according to the international standard 10/20 system, with additional electrooculogram (EOG), mental/submental electromyogram (EMG) and a two-lead electrocardiogram (ECG) (32 channels cap modified for sleep recordings, Easy Cap, Herrsching-Breitbrunn, Germany), referenced against FCz. Data were continuously sampled throughout the experiment at 5 kHz. To allow for optimal artifact correction, EEG recordings and fMRI were synchronized using the scanner's 10 MHz master clock [31]. Electrode impedance was below 5 kΩ Raw data were stored without any filtering for later processing. In addition, trigger pulses from the MRI system as well as from the stimulus presentation device were recorded for subsequent off-line artifact correction and identification of tone events, respectively.

### EEG data analysis

#### Data preprocessing and identification of K-complexes

Data were analyzed with Brain Vision Analyzer, Version 1.05 (Brain Products, Gilching, Germany). EEG recordings were corrected for gradient artifacts using the implemented algorithm based on subtraction of adaptive artifact templates, and downsampled to 250 Hz. To remove pulse artifacts, EEG signals were transformed using independent component analysis (ICA), and components with activity synchronous to the ECG recording were excluded prior to back transformation. EEG traces were re-referenced against linked mastoid electrodes. EEG data were subsequently band-filtered (0.5–70 Hz; EOG: 0.1–30 Hz, EMG: 16–250 Hz), with an additional notch filter set to 50 Hz. The corrected polysomnographic recordings were analyzed using visual scoring of sleep stages according to the Rechtschaffen & Kales criteria (1968). For each subject, a continuous episode with steady sleep stage 2 was selected from the complete fMRI time series for further analysis. KCs were then identified visually as strictly biphasic waves of a total duration>500 ms, starting with a negative wave (500 to 800 ms following stimulus onset [1]), immediately followed by a positive deflection. As an amplitude criterion, the negative-positive deflections had to reach voltage differences of at least 50 µV (instead of 75 µV [32] since ERPs have been shown to be reduced in amplitude in the fMRI environment [33]). Similar to Colrain et al. [32], other evoked responses with increased amplitudes (>50 µV as described in literature; >35 µV to account for the fMRI environment) but not fulfilling the criteria for a KC (also referred to as unspecific responses) were also identified but excluded from further analysis. This latter category may include vertex sharp waves, delta waves and other low frequency components. For wakefulness, same EEG preprocessing steps were performed.

#### Analysis of evoked potentials

Averages of evoked potentials were calculated (time window of −200 ms to 1800 ms) with the tone onset defined as time 0 ms, separately for epochs following frequent and rare tones. Furthermore, rare epochs in sleep stage 2 were subdivided into trials with and without evoked KCs (rare_KC_ and rare_w/oKC_, respectively). A baseline correction was applied (baseline −200 to 0 ms), followed by DC detrending of the EEG signal. Finally, the resulting EEG traces were averaged for each individual channel using the three event types: frequent, rare_KC_ and rare_w/oKC._ Distinct local maxima were labeled according to their latency, following the classification by [1]: N1 as negative peak between 90 and 110 ms, P2 as positive peak between 170 and 230 ms, N350 as negative peak between 250 and 400 ms, P450 as positive peak between 360 and 540 ms, and N550 as negative peak between 500 and 800 ms. For each subject, the maximum amplitude of the averaged responses in the respective time window was extracted for rare_KC_, rare_w/oKC_ and frequent tones for the central electrodes Fz, Cz and Pz. Statistical analysis of amplitudes comprised each two factorial analysis of variance (3-level-factors electrode and trial-type) for the N1, P2, N350, P450 and N550 component. Main effects and the electrode×trial-type interaction were analyzed and post-hoc (LSD) tests applied if appropriate. The MMN, involved in passive deviant detection, is best observed in a subtraction waveform of rare and frequent tone averages. The MMN has been reported to occur in this subtraction waveform as a negative peak between 100–250 ms after tone presentation [34]. Due to the effective sampling rate of 250 Hz (corresponding to 4 ms resolution) in our EEG data after preprocessing, we have chosen the post-stimulus window 108–260 ms for MMN analysis, subdivided into three frames of 48 ms each. The average of the subtraction waveform in the 48 ms prestimulus period served as zero amplitude baseline. For each frame, the minimum amplitude was determined, likely reflecting the MMN, and subjected to one-tailed t-tests (with negative directionality assumed).

#### Analysis of stimulation-induced EEG frequency shifts

To interrogate if rare tones or KCs induce changes in sleep depth, particularly arousal reactions, we employed EEG frequency analysis of periods preceding and following the rare tone presentations. For this, spectral power of the delta (1–4 Hz), theta (4.5–7.5 Hz), alpha (8–11 Hz), sigma (11.5–16 Hz) and beta (16.5–25 Hz) frequency bands were compared between frequent epochs before (frequent_pre_) and after (frequent_post_) each rare epoch with the time window defined to 100–1100 ms after frequent tone presentation. For each channel and frequency band, the difference (frequent_post_–frequent_pre_) of spectral power induced by the rare tone was tested for its deviation from zero (10 subjects, one-sample t-test). The group t-test was performed for all rare tones (rare_KC_ and rare_w/oKC_) and separately for rare_KC_ and rare_w/oKC_. In analogy to the ERP analysis, events with unspecific slow responses were excluded.

#### EEG components of no interest

Only few KCs showed subsequent visually identifiable sleep spindles [35] in our experiment. To avoid any contamination by possibly KC-induced activity in individual segments, we introduced alpha and spindle activity as regressors of no interest in the later fMRI models. To do so, FFT based spectral power of the alpha (8–11 Hz, at position O1 and O2) and spindle frequency band (11.5–16 Hz, at position Cz and Fz) were extracted from segments of 2 s length (overlap of 1 s). For each frequency band, power values were summed up and assigned to the middle of the respective temporal window.

### fMRI data analysis

All MR analysis steps were performed on Linux workstations using the SPM software (http://www.fil.ion.ucl.ac.uk/spm, version SPM5). First, data were corrected for slice time differences to compensate for different acquisition times due to the interleaved slice acquisition scheme in each volume. Afterwards, a realignment step was performed using rigid body transformation. Movements less than 2 mm were accepted. Data were then normalized to a standard EPI template in MNI space (SPM5 distribution), resliced to a voxel resolution of 2×2×2 mm^3^ (5^th^ degree spline interpolation), and smoothed using an isotropic Gaussian kernel with full width half maximum of 8 mm. As mentioned above, only periods showing continuous sleep stage 2 were considered for further analysis.

Tone trials were separated into seven regressors representing rare tones evoking i) a KC (rare_KC_), ii) another response type (rare_X_), iii) no obvious high amplitude EEG response (rare_w/oKC_); frequent tones evoking iv) KCs (frequent_KC_), v) another response type (frequent_X_), vi) no high amplitude EEG response (frequent_w/oKC_); and vii) KCs not directly associated to any tone (N550 component>0.8 s after preceding tone presentation. Regressor vi) was orthogonalized with respect to all other regressors (i–v; vii). Beside regressors of interest, the model comprised the following set of nuisance regressors (NR): NR1 and NR2: total white matter intensity and cerebrospinal fluid (CSF) intensity per fMRI volume. NR3 and NR4: Alpha power (8–11 Hz) and spindle power (11.5–16 Hz) extracted as described above and convoluted by the HRF. NR6–NR11: parameters of the rigid body motion correction, and NR12–NR17: Differential motion correction parameters to account for relative motion between the i^th^ and (i-1)^th^ volume.

Positive and negative T-contrasts were evaluated for all rare tones (rare_KC_, rare_w/oKC_, and both regressors combined), as well as for the differential contrast (rare_KC_ – rare_w/oKC_).

#### Second level analyses

2^nd^ level random effect analyses were performed across subjects for sleep trials (one sample t-tests). When comparing all rare tone against frequent tones significance was set to FDR corrected p_FDR_<0.05, extent 10 voxels, for both wakefulness and sleep stage 2. A mask defining task specific activation in wakefulness was built using this threshold. For sleep stage 2, significance in the differential contrast rare_KC_ vs rare_w/oKC_ was accepted for p-values<0.001, uncorrected, and an extent threshold of 10 voxels. Anatomical location of significant clusters was determined from comparison with a canonical T1-weighted image in MNI space and by automated assignment of clusters to Brodmann areas and brain regions in MNI space (http://www.fil.ion.ucl.ac.uk/spm/ext/#MSU). To evaluate task specific activation during sleep the mask described above was applied and activated voxels were collected at a threshold of p<0.05. These latter results were presented as corrected p-values on a cluster level (p_cluster_).

## Results

All 18 subjects were included for the analysis performed during wakefulness. For the sleep trial, one participant was not able to fall asleep at all and six participants only reached sleep stage 1 or spent less than 3 minutes continuously in stage 2. Another participant showed movement artifacts outside the tolerated range during the sleep recording. These subjects were excluded from further analysis. Finally, data from ten subjects (5 m/5 f, mean age 25.5±2.7 years) were used for further fMRI/EEG analyses focusing on sleep stage 2. Of these ten subjects, two subjects reached sleep stage 1 after less than one minute, three subjects after 3 minutes and five subjects after more than 5 minutes. Consolidated sleep stage 2 was reached after a mean latency of 10′13″±5′55″ min (range 0′44″–17′39″ minutes). fMRI volumes were extracted for continuous episodes in sleep stage 2 with an average duration of 14′55″±6′0″ minutes (range 8′54″–26′0″ minutes).

### Appearance of KCs

Within selected sleep stage 2 fMRI episodes, we observed a median of 31 well-defined KC complexes (range 19–160) with an average density of 3.4±1.8 KCs/min. KCs evoked by tones occurred with a median of 21 (range 9–125). Of these, a median of 16 KCs (range: 5–109) were elicited by rare tones, while a median of 6 KCs (range: 4–16) occurred after frequent tones. As compared to all rare and frequent tones presented per individual this reflects an appearance of KCs in 23% of rare tones (range: 5–75%) and 2% of frequent tones (range: 1–4%), respectively. In addition, a median of 33 (range: 10–71) unspecific responses (2.4±0.87/min) were identified. Finally, a median of 13 KCs (range: 1–35) arose which where not related to tone presentation, i. e. appearing outside the above described temporal detection window.

### Event-related potentials (ERP)

**Figure 1:**
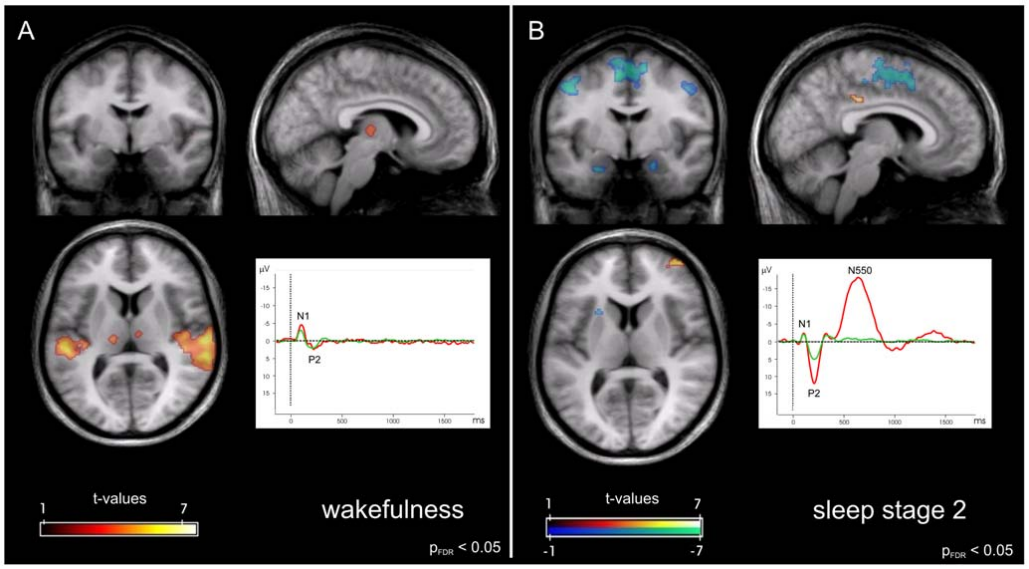
Neuronal responses to rare tones as compared to frequent tones. Comparison between wakefulness (A) and sleep stage 2 (B). Orthogonal views at MNI coordinates x = 7, y = −5, z = 7. Inserts show ERPs to frequent (green) and rare tones (red) at electrode position Cz. FMRI data are thresholded at p_FDR_<0.05, cluster extent>10.

Comparing rare and frequent tones during wakefulness, ERP amplitudes were significantly larger for P300 (p<0.001) following rare tones, but not for N1 and P2 (Figure 1A). A significant effect of EEG channel was found for P300, with higher amplitude in more parietal electrodes. Similar, a significant effect for MMN was found in Pz in the time window 160–208 ms (−0.861±2.025 µV; p = 0.022), as expected for pre-attentive stimulus discrimination (see Figure S1).

As described in [1], ERPs during sleep showed a decrease in N1 amplitude, an increased amplitude of P2, as well as appearance of a small N350 and a large-amplitude N550, the latter representing the KC (Figure 1B). During selected sleep stage 2 epochs, differences of ERP amplitudes between trial types (rare_KC_, rare_w/oKC_, frequent) were significant for components N1, P2, P450, N550 and P900, all p-values<0.001, and non-significant for N350 and MMN. All components in rare_KC_ trials were higher in (absolute) amplitudes than in frequent or rare_w/oKC_ trials, and rare_KC_ differed significantly in P2, N550 and P900 from the other two trial types. Post-hoc comparisons demonstrated that for P450, also rare_w/oKC_ trials differed significantly from frequent trials. The difference between rare_w/oKC_ and frequent trials was absent for the P2, N550 and P900 response, highlighting specificity of the latter components for KCs.

**Figure 2:**
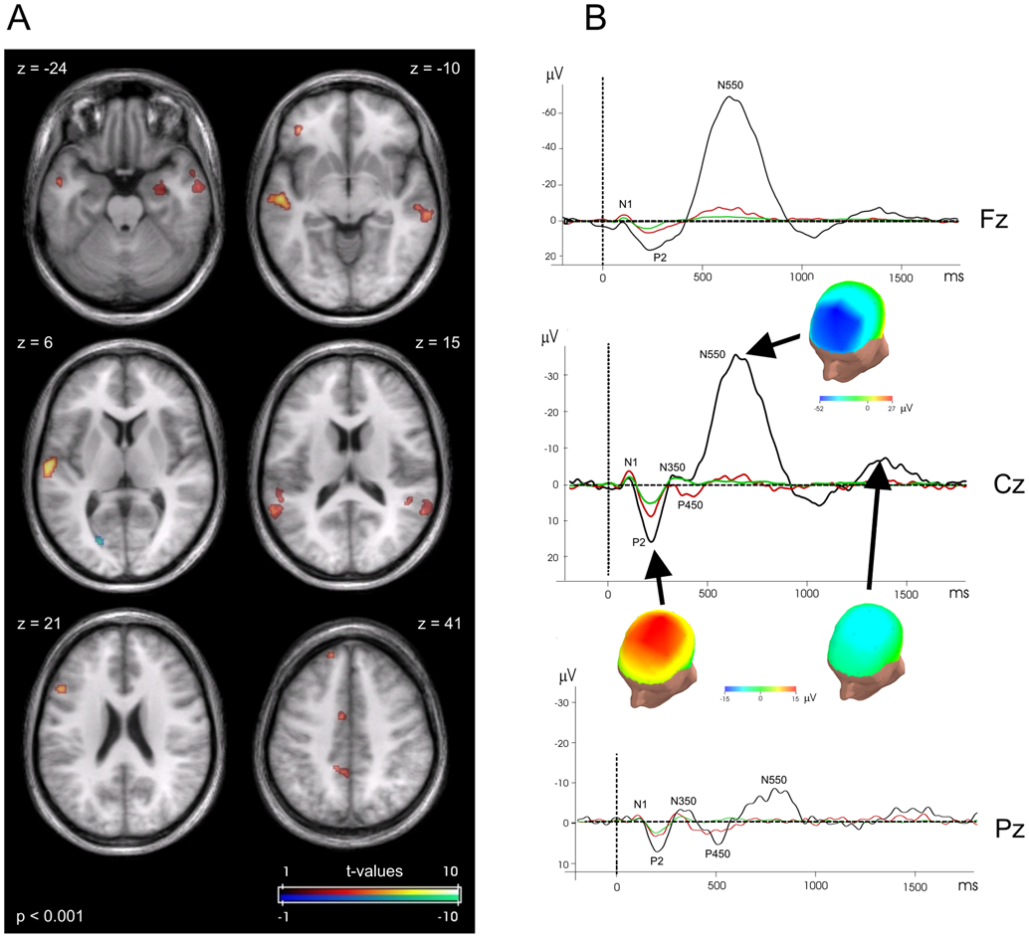
Differences in activation patterns dependent on evoked K-complexes. (A) Comparison of activation for rare tones followed by a KC against rare tones not evoking a KC response. Axial slices are indicated with MNI z-coordinates. FMRI data are thresholded at p_uncorr_<0.001, cluster extent>10 voxels. (B) Evoked potentials for the Fz, Cz and Pz derivation. Green: frequent tones; red: rare_w/oKC_ tones; black: rare_KC_ tones. Surface mapping of EEG amplitudes for selected components is inserted.

Electrode×trial type interactions were found for all analyzed components, reflecting generally higher sensitivity for the more anterior electrodes for amplitude differences (Figure 2B).

### Effect of rare tones on sleep depth

For all rare tones, spurious spectral power changes were detected in a limited number of frequency bands at a significance threshold of 0.05 (uncorrected; P7: delta increase; O2: theta decrease and beta increase), not robust against multiple test correction. When separated by the appearance of a KC, no changes of frequency bands were detected for rare_w/oKC_, and spurious changes, mostly increases, of theta (O2, F8, T8 and P7) and delta (O2) activity for rare_KC_, again not robust against multiple test correction. No changes were detected in either analysis for the alpha and sigma frequency band at uncorrected p<0.05.

### fMRI results

#### Passive oddball task during wakefulness and S2

The overall response to the passive oddball task during wakefulness is given in Figure 1A (detailed in Table 1), along with the grand average ERPs for rare and frequent tones measured at the Cz derivation. During wakefulness, positive BOLD responses indicating activations were detected bilaterally in regions also described for active oddball paradigms [36], namely the auditory cortex and thalamus.

**Table 1:** Activation contrasting odd tones against even tones during wakefulness.

		Brain Region	Brodmann areas, deep nuclei	Cluster size (voxel)	Z score	x	y	z
		Positive BOLD responses						
1	L	Middle/superior temporal gyrus, insula	13,21,22,29, 40,41,42	943	5.67	−54	−30	12
2	R	Middle/superior temporal gyrus	13,21,22,41,21	2293	5.31	64	−36	10
3	L	Thalamus	VPMN, MDN, Pulvinar, LPN	33	3.71	−14	−20	8
4	R	Thalamus	MDN	37	3.41	6	−18	6
5	R	Superior temporal gyrus	38	13	3.35	38	4	−16

Clusters resulting from second level random effects analysis (t test, p*_FDR_*<0.05). Regions showing significant activation to the odd tones as compared to even tones are listed. Sorting is after Z-values of the cluster peak voxel. Brodmann areas are identified for clusters covering>3% of the respective area. Coordinates (x, y and z) are given in MNI space. MDN: Medial dorsal nucleus, VPMN: ventral posterior medial nucleus, LPN: Lateral posterior nucleus.

For sleep stage 2, significant clusters in response to the rare tones are shown in Figure 1B and Table 2, along with the grand average ERPs measured at the Cz derivation. Here, no activation of the auditory cortex (Brodmann areas 22, 41 and 42) nor of the thalamus as in wakefulness could be observed in either the whole brain analysis or after restriction to the task-specific mask (p_cluster_>0.95 for all regions of interest). Regions showing increased activity were located in the bilateral middle temporal gyrus and superior parietal lobule, right middle and superior frontal gyrus and posterior cingulate cortex, and in the left precuneus.

**Table 2:** Activation contrasting odd tones against even tones in sleep stage 2.

		Brain Region	Brodmann areas, deep nuclei	Cluster size (voxel)	Z score	x	y	z
		Positive BOLD responses						
1	R	Middle temporal/occipital gyrus	19,37,(21)	415	5.44	50	−44	−12
2	L	Parietal lobule/precuneus	7, 40	1053	5.14	−34	−60	36
3	R	Inferior parietal lobule	7, 40	711	4.35	42	−56	32
4	R	Middle/superior frontal gyrus	10	47	4.25	40	56	22
5	R	Middle/superior frontal gyrus	10	163	4.14	34	58	2
6	L	Inferior temporal/middle occipital gyrus	37	159	4.06	−46	−68	−6
7	R	Cingulate gyrus	23, 31	18	3.96	6	−32	36
8	R	Middle/superior frontal gyrus	8	71	3.96	32	26	46
9	L	Superior parietal lobule	7	21	3.87	−12	−70	60
10	L	Middle temporal gyrus	37	58	3.77	−46	−52	−14
11	L	Angular/supramarginal gyrus	39, 40	32	3.41	−54	−68	34
12	L	Precuneus	7	30	3.41	−8	−58	38
		**Negative BOLD responses**						
13	L	Precentral gyrus, middle frontal gyrus	4,6,9	333	5.22	−50	2	42
14	R/L	Medial frontal, superior frontal (R) gyrus	6,24,32	1435	5.08	−14	2	70
15	L	Lentiform nucleus, claustrum	Putamen	112	4.37	−24	14	0
16	L	Precentral/middle frontal gyrus	6	11	4.00	−32	−12	50
17	L	Inferior frontal gyrus	34	101	3.96	−32	2	−4
18	R	Parahippocampal/inferior frontal gyrus, extra-nuclear	Amygdala, 13, 47	129	3.88	30	8	−14
19	L	Parahippocampal gyrus	Amygdala	35	3.67	−22	−2	−26
20	R	Cuneus	17	24	3.66	16	−94	0
21	R	Paracentral lobule	31	11	3.56	8	−24	50
22	R	Middle frontal gyrus	6	11	3.56	28	2	52
23	R	Precentral gyrus	4,6	62	3.49	52	−8	44
24	R	Middle frontal gyrus	6	14	3.48	50	6	56
25	R	Precentral gyrus, inferior/middle frontal gyrus	6,9	36	3.39	42	−2	38

Clusters resulting from second level random effects analysis (t test, *p_FDR_*<0.05). Regions showing significant (de-)activation to the odd tones as compared to even tones are listed. Sorting is after Z-values of the cluster peak voxel. Brodmann areas are identified for clusters covering>3% of the respective area. Coordinates (x, y and z) are given in MNI space.

Furthermore, NBR to rare tones was found bilaterally in the amygdala, precentral and frontal gyrus, as well as in the right cuneus and the left putamen.

#### Differential effect of KCs following rare tones

Differential response to rare tones in sleep stage 2, depending on the presence or absence of subsequent KCs (i. e. rare_KC_ versus rare_w/oKC_), was assessed. Here, stronger BOLD response to rare_KC_ was found bilaterally in the auditory cortex (BA 21,22) and adjacent areas (masked task-specific clusters: left auditory cortex p_cluster_ = 0.149; right p_cluster_ = 0.019) (Table 3 and Figure 2A). Activation was also increased in the left precuneus (BA 31), ventral anterior cingulate cortex (BA 24), and left dorsolateral frontal gyrus (BA 9,46). Furthermore, we observed increased activity in the bilateral superior temporal gyrus (BA13) and the right hippocampus. A single cluster in the cuneus (BA 17) showed a NBR in this contrast. Notably, we only observed limited positive BOLD contrast in superior fronto-central brain regions in which we hypothesized strongest reflection of KCs (Table 3). To increase the sensitivity on potentially delayed fMRI responses associated with the extend duration of the KC itself, we estimated our model with extention of the stimulus duration from 0 ms to 1500 ms. Doing so, the full length of the KC, including the N550 and P900 deflections, was modeled. The results basically highlighted the same areas as for the event related differential contrast applying 0 ms duration. Some areas in dorsal regions show slightly increased extent (see Table S1).

**Table 3:** Activation contrasting odd tones with evoked KCs against odd tones without evoked KCs in sleep stage 2.

		Brain Region	Brodmann areas, deep nuclei	Cluster size (voxel)	Z score	x	y	z
		Positive BOLD responses						
1	L	Middle frontal gyrus	47	19	4.80	−40	40	−8
2	L	Middle/superior temporal gyrus	21,22	460	4.59	−62	−18	8
3	R	Inferior/middle temporal gyrus	21	52	3.72	64	−6	−20
4	R	Superior temporal gyrus	22	56	3.72	64	−52	12
5	L	Superior temporal gyrus	22	36	3.68	−60	−50	14
6	R	Parahippocampal gyrus	Hippocampus	21	3.66	30	−10	−24
7	R	Middle temporal gyrus	21	13	3.64	52	4	−26
8	L	Cingulate gyrus	24	19	3.63	−6	0	42
9	R	Middle temporal gyrus	21	55	3.60	64	−30	−8
10	L	Cingulate gyrus/precuneus	7,31	46	3.35	−8	−48	36
11	L	Superior temporal gyrus	13	19	3.34	−56	−36	16
12	R	Superior temporal gyrus	13	13	3.33	48	−44	14
13	L	Superior frontal gyrus	8	15	3.58	−14	50	44
		**Negative BOLD responses**						
14	L	Cuneus	17,30	19	4.97	−20	−78	6

Clusters resulting from second level random effects analysis (t test, *p_uncorr_*<0.001). Regions showing significant (de-)activation to the odd tones evoking a KC response as compared to odd tones without KCs are listed. Sorting is after Z-values of the cluster peak voxel. Brodmann areas are identified for clusters covering>3% of the respective area. Coordinates (x, y and z) are given in MNI space.

Averaged ERPs for the midline electrodes (Fz, Cz and Pz) are given for comparison in Figure 2B, along with the projection of the respective ERP amplitudes on a head model. These data reassured a frontocentral maximum of the N550 deflection in our simultaneous EEG/fMRI setup.

## Discussion

The present study combined fMRI/EEG to analyze the cerebral responses to auditory stimulation in light NREM sleep. Using an acoustic oddball paradigm, we revealed a prominent negative BOLD response (NBR) for rare tones compared to the background of frequent tones. This NBR occured independently of the generation of evoked KCs and was found predominantly in the motor cortex, the premotor and the supplementary motor areas, the bilateral dorsomedial PFC, and the bilateral amygdala. No wake-like activation of the auditory cortex was detected. Rare tones followed by an evoked KC, however, were associated with a wake-like activation of task-related areas in the temporal cortex, along with the right hippocampus.

The current study extends earlier findings on stimulus-induced NBR and its correlation with the increase in delta power during light NREM sleep [26], [27]. In contrast to our former report, no EEG frequency changes immediately after the rare tones were detected in the present study. Especially, no increase in higher frequency bands, an indicator of arousal, was found, and rather spurious slowing in the subsequent frequent epoch was observed. These differences to our earlier report can be attributed to the experimental design: The former data were based upon a paradigm with continuous stimulus presentation during blocks of 30 seconds, and the observed NBR and slowing of the EEG likely reflected habituation and longlasting sleep protective mechanisms. In the current study we used an event-related design which has stronger arousal capacities and which involves detection of tone deviance and related reflexive orienting responses. Still, the simultaneous EEG recordings confirmed stability of the sleep stage during the experiment.

During wakefulness, the detection of rare tones was accompanied by a MMN consistent with pre-attentive discrimination processes [34], [37]. An increased P300 was also observed, however, with rather small amplitude as compared to previously reported findings in active oddball paradigms [34], [37]. The fMRI activation pattern found for rare tones in wakefulness resembles the previously reported activation for the passive oddball paradigm, including predominantly superior temporal areas [38], [39]. In line with previous findings, P300 was not detectable in sleep stage 2, and sleep specific late ERP components arose [2], [40]. Furthermore, absence or strongly reduced amplitude of a MMN component has frequently been described during light NREM sleep [3], [6], [7], [41], suggesting that auditory processing is strongly affected during NREM sleep.

Noteworthy, the sleep-specific NBR was most pronounced in areas related to voluntary movements. We therefore submit that the transient suppression of brain areas involved in the generation and execution of motion activity is one of the factors that allows further consolidation of sleep. Reduced activity in the amygdala points towards altered processing of the emotional salience of the incoming stimulus during sleep [42]. In the passive oddball condition during wakefulness, the amygdala generally does not show specific activation for rare tones [43]. However, it was shown earlier that unpredictable irregularities in acoustic stimulus series as well as rare tones embedded in trains of frequent tones may lead to activation of the amygdala [44]. The amygdala has also been reported to constitute part of a network activated in the active oddball paradigm, both for rare and deviant tones [36]. Amygdala activation, however, is not prerequisite for target detection as demonstrated from lesion studies in epileptic patients [45]–[47]. Rather, it was suggested that salient stimuli lead to a reflexive orienting response with consequent preparative engagement of additional brain regions, including the amygdala, for the case of further sensory income [36]. During wakefulness, such an orienting reflex has been shown to be associated with an increased level of arousal, as reflected by EEG desynchronization, increased skin conductance and modulation of heart rate [48]. During sleep, autonomous arousal reaction may be suppressed in order to maintain and consolidate sleep. Accordingly, our EEG analysis during sleep did not reveal signs of such arousal reactions. Thus, we interpret the NBR in the amygdala as a sleep specific response, and submit that it reflects suppression of a reflexive orienting responses to the non-alarming, but unpredictable rare tone, supporting sleep protection by delimiting arousal reactions. We did neither observe activation of the auditory pathway nor the thalamus, in line with our previous work [26], [27] and data shown by Tanaka et al. in sleep stage 1 [25]. The opposed findings of Portas and colleagues [22], who reported preserved activation of the auditory cortex and thalamic regions during light NREM sleep, may originate from the use of more salient, especially personal stimuli such as the own names. Awakenings are highly likely to occur upon such stimuli and have indeed been reported by the authors for 30–50% of trials [22]. In addition, transient arousals during fMRI data acquisition may have remained undetected as EEG signals during scanning were not evaluated.

When segregating the rare tone with respect to appearance or non-appearance of an evoked KCs as the brain's prominent EEG response upon external stimulation, we found activation in the bilateral auditory cortex and the left middle and superior frontal cortex, precuneus, posterior cingulate, bilateral superior temporal cortex and the right hippocampus. As a whole, this network largely overlapped with the response to a novel (task-irrelevant) stimulus as compared to rare tones during wakefulness [36], with exception of the cingulate cortex and the right hippocampus. As anticipated by Sallinen [17], the sleeping brain may in fact be transiently more responsive to sensory events preceding a KC than preceding a non-KC response to a stimulus. The similarity of our results with the novelty oddball paradigm suggests an orienting response, in which the stimulus characteristics of the rare tone undergo a re-evaluation during sleep. This view is supported by a recent study reporting direct intrahippocampal ERP measurements in 16 epilepsy patients during a passive oddball paradigm. This study revealed involvement of hippocampal and parahippocampal structures in the preattentive processing of sound deviance during wakefulness [49]. Since the hippocampal potentials were shown to be reduced upon repetitive presentation of the rare tones, the authors proposed that they may be regarded as a central marker of an orienting response, facilitating the detection of sound deviance when sound discrimination becomes more difficult. Hippocampal and temporolateral activation as observed in our study may signify an update of stimulus characteristics during sleep. In summary, the general response to rare tones is reduced during sleep, however, a transiently more responsive brain state appears to occur in association with the generation of KC.

The precuneus, obviously playing a pivotal role in integrated tasks, reduces its activity during sleep [50], [51]. Thus, increased activity in the precuneus (and cingulate cortex) in response to rare tones eliciting KCs may be conceived as an additional signature of intensified stimulus processing.

Furthermore, we only observed BOLD activation in the superior frontocentral regions associated with KCs in a small number of areas. Given that KCs are the strongest of any evoked potentials recorded in humans [1], the finding that rare_KC_ events do not show a stronger correlate in the BOLD signal as compared to rare_w/oKC_ warrants further discussion. The strong topographical similarity of the NBR for both rare_KC_ and rare_w/oKC_ tones suggests common responses to the stimuli at the level of fMRI as e. g. reflected by the early N350 component with maximal values over vertex regions [32]. Bastien and Campbell [52] discussed whether evoked KCs are an all-or-none phenomenon: An external stimulus either elicits a KC or it does not, as averaging trials not showing an N550-P900 amplitude difference of at least 75 µV did not reveal any evidence of a N550 deflection at all. It was suggested that 'although the KC may occur in association with other indices of a defensive response, defensive responses also occur in the absence of the KC' [52]. Sleep defensive responses upon external stimulation as reflected by heart-rate acceleration was reported by Church et al. [53] to be independent of the occurrence of KCs [54]. This notion is now further supported by the present combined fMRI/EEG measurements that revealed an NBR response upon tone presentation, irrespective of the appearance of an evoked KC.

The discrepancy of a small difference in NBR between rare_KC_ and rare_w/oKC_ and a strong electrophysiological difference may further be related to the degree of synchronicity of the firing of cortical areas. While response to both types of rare tones may be associated with comparable metabolic demands, the electrophysiological response may not mount to a surface KC in the case of asynchronous firing. A recent computational study simulated KCs in terms of a mean field cortical model [55]. The authors interpreted the appearance of a KC as a momentary excursion of the cortex from a stable low-firing state to an unstable sleep specific high-firing state upon a small excitation. They observe a critical threshold for external disturbances, above which KCs are evoked, in accordance with the ‘all-or-none’ hypothesis. The momentarily increased stimulus transmission and processing may allow external stimuli to exceed this critical cortical threshold and may eventually lead to initiation of an evoked KC. However, our data suggest that the overall metabolic cerebral demand in regions showing highest EEG deflection during KCs is not strongly altered when the threshold for a KC is exceeded.

KCs have been postulated to have a diffuse cortical generator and represent a sleep specific, but modality independent response. This response does not depend on activation of the auditory pathway in the case of acoustic stimulation, or activation of the primary sensory pathway when elicited by inspiratory occlusion [14]. Therefore, increased activation of task-specific auditory pathways associated with evoked KC is unlikely to reflect the neural representation of the KC itself. Future studies should test this hypothesis by applying stimuli of different sensory modalities during NREM sleep fMRI experiments. Another option may be to target spontaneous KCs in undisturbed sleep without any external stimulation. However, to observe truly spontaneous KCs is technically demanding due to the noise during fMRI experiments.

Finally, our data only allow an indirect interpretation of the functional significance of KCs. We found that a brain state in which stimuli provoke an intensified brain activation (most closely resembling active novelty processing) is prone to generation of subsequent KCs. However, the passive oddball data collected during wakefulness do not directly support this notion. Still, it should be noted that the passive listening condition during wakefulness and the ‘passive’ condition of the subject while falling asleep and during established NREM sleep have categorically different qualities: During wakefulness, the subject retains awareness and can be instructed not to focus on deviant tones, while during sleep conscious control as necessary to follow task instruction cannot be maintained. Therefore, responses become automated and independent of any instruction. As these automated processes take over during sleep, external stimuli may be sporadically (re-)evaluated with respect to the level of novelty or deviation from the background. Our data show that rare tones followed by a KC exhibit signatures of increased activity, comparable with novelty processing as described by Kiehl et al. [36] during wakefulness. However, since EEG analysis did not reveal signs of arousal, we propose that the subsequently evoked KCs may rather reflect maintainance and consolidation of sleep than an arousal reaction.

The refractory period of KCs is roughly 10 seconds. Our design using an ISI of 2 seconds was optimized to measure KCs evoked by rare tones with a probability of 20%, thus roughly matching the refractory period. We intentionally avoided longer ISIs due to potential arousing effects of sparsely delivered tones. As a drawback of our design, we did not collect a sufficient number of KCs evoked by frequent tones, or spontaneous KCs, for fMRI analysis comparing different types of KCs. Regarding activation changes related to individual ERP components, the temporal resolution of BOLD signal changes in the order of several seconds did not allow to dissect the contribution of individual ERP components in our sample. Even more, if N550 and P900 represent opposing up and down states, the individual contributions may lead to a destructive interference of BOLD signal changes on the time-scale of fMRI measurements. Unfortunately, the limited number of KCs in our data do not allow to dissect contributions of these two ERP components. Further studies with larger numbers of KC events are required to disentangle possible different functional brain states associated with individual ERP components.

It has been shown earlier that the scanner noise leads to reduced ERP amplitudes, prolonged reaction times and decreased hit rates in active oddball paradigms [56], [57]. Generally reduced amplitudes in the electrophysiological measures during fMRI due the main magnetic field [33] have been accounted for by lowering the absolute amplitude threshold for identification of KCs during sleep. Otherwise, the influence of the scanner sound was considered to introduce similar reductions in ERP amplitudes during both wakefulness and sleep, still allowing for comparison of the respective ERP signals. However, our observations of rather small P300 amplitude differences between rare and frequent tones during wakefulness may in part be related to interference with the fMRI background noise.

Due to reduced quality of fMRI data caused by susceptibility artifacts in ventral frontal brain regions, we cannot fully exclude that such artifacts might delimit detection of KC related activity in ventral brain areas. However, as we observed significant activation changes in other basal brain regions like amygdala and hippocampus, a total loss of fMRI information in ventral frontal regions seems unlikely.

In conclusion, our data indicate altered acoustic processing during light NREM sleep in an oddball paradigm, showing strongly reduced activity of the auditory system in response to rare tones. We interpret a negative BOLD response in sensory motor areas and the amygdala as a sleep defensive mechanism delimiting reflexive orienting responses. Events followed by evoked KCs revealed increased activation of the auditory system and hippocampal and temporal structures probably reflecting increased responsiveness to the external stimuli and an updating of stimulus characteristics. However, other than expected, the strong electrophyiological signature of the K-complexes in frontocentral areas was not paralleled by corresponding BOLD increases. This dissociation between electrophysiological and metabolic correlates of KCs argues for increased cortical synchronization without additional metabolic demand.

## Supporting Information

Table S1 Activation contrasting odd tones evoking KCs vs odd tones without evoked KC in sleep stage 2. Assumed stimulus duration 1500 ms. Clusters resulting from second level random effects analysis (t test, puncorr<0.001) of the model assuming a event duration of 1500 ms. Regions showing significant (de-)activation to the odd tones evoking a KC response as compared to odd tones without KCs are listed. Sorting is after Z- values of the cluster peak voxel. Brodmann areas are identified for clusters covering>3% of the respective area. Coordinates (x, y and z) are given in MNI space.
(0.04 MB DOC)

Figure S1 Mismatch negativity analysis. Subtraction waveform of rare and frequent tone averages, as used for analysis of MMN, measured at Pz. Green curve: wakefulness. Red and black curves: sleep stage 2, separated according to responses without and with evoked KCs, respectively. Position of the MMN in wakefulness is indicated. Negative deflections at 100 ms during sleep may be attributed to changes in N1 amplitude rather than occurance of a MMN during sleep.
(5.75 MB TIF)
