# Supplementary material for: Acoustic Oddball during NREM Sleep: A Combined EEG/fMRI Study
Source: PLoS One. 2009 Aug 25;4(8):e6749. doi: 10.1371/journal.pone.0006749 (PMC2727699; doi:10.1371/journal.pone.0006749)
Supplement: Table S1 — Activation contrasting odd tones evoking KCs vs odd tones without evoked KC in sleep stage 2. Assumed stimulus duration 1500 ms. Clusters resulting from second level random effects analysis (t test, puncorr<0.001) of the model assuming a event duration of 1500 ms. Regions showing significant (de-)activation to the odd tones evoking a KC response as compared to odd tones without KCs are listed. Sorting is after Z- values of the cluster peak voxel. Brodmann areas are identified for clusters covering>3% of the respective area. Coordinates (x, y and z) are given in MNI space. (0.04 MB DOC) [file pone.0006749.s001.doc]

**Table S1 – Activation contrasting odd tones evoking KCs vs odd tones without evoked KC in sleep stage 2. Assumed stimulus duration 1500ms.**

|  |  | **Brain Region** | **Brodmann areas, deep nuclei** | **Cluster size (voxel)** | **Z score** | **x** | **y** | **z** |
| --- | --- | --- | --- | --- | --- | --- | --- | --- |
|  |  | **Positive BOLD responses** |  |  |  |  |  |  |
| 1 | R | Middle frontal gyrus | 47 | 23 | 5.06 | -40 | 42 | -8 |
| 2 | L | Middle/superior temporal gyrus | 21,22 | 343 | 4.50 | -54 | -16 | -8 |
| 3 | L | Cingulate gyrus | 24 | 37 | 4.08 | -8 | 0 | 44 |
| 4 | R | Superior temporal gyrus | 22 | 49 | 3.97 | 64 | -52 | 12 |
| 5 | L | Inferior frontal gyrus | 45 | 24 | 3.82 | -52 | 20 | 20 |
| 6 | R | Insula | 13 | 22 | 3.81 | 34 | -2 | 14 |
| 7 | R | Middle temporal gyrus | 21 | 11 | 3.68 | 50 | 6 | -28 |
| 8 | R | Inferior/middle temporal gyrus | 20,21 | 22 | 3.62 | 64 | -6 | -22 |
| 9 | L | Cingulate gyrus | 7,31 | 58 | 3.59 | -4 | -46 | 40 |
| 10 | L | Superior frontal gyrus | 8 | 26 | 3.58 | -14 | 48 | 44 |
|  |  | **Negative BOLD responses** |  |  |  |  |  |  |
| 11 | L | Cuneus | 17,30 | 17 | 4.06 | -22 | -74 | 4 |
