# Supplementary figures and images for: Acoustic Oddball during NREM Sleep: A Combined EEG/fMRI Study
Source: PLoS One. 2009 Aug 25;4(8):e6749. doi: 10.1371/journal.pone.0006749 (PMC2727699; doi:10.1371/journal.pone.0006749)

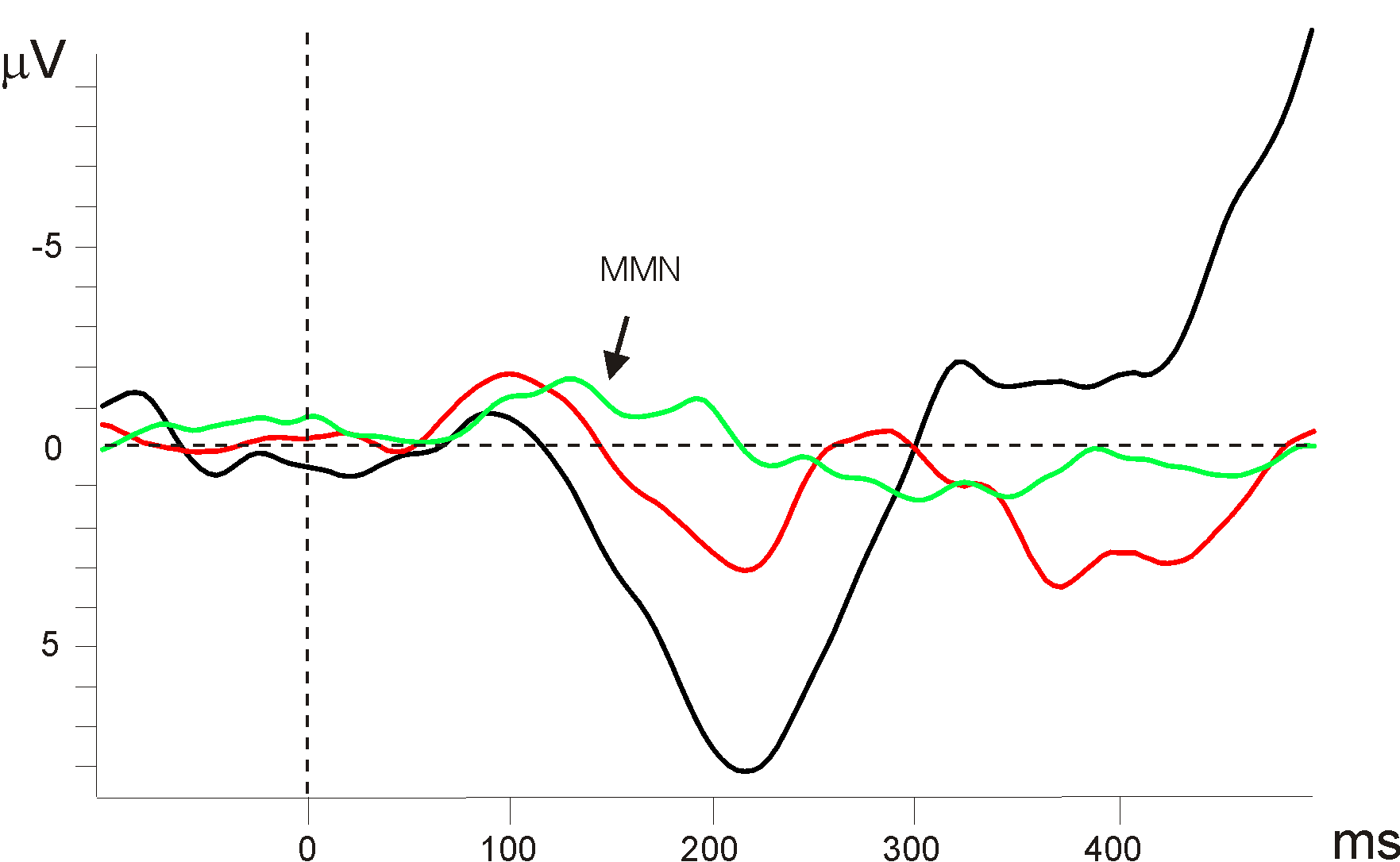

Supplement: Figure S1 — Mismatch negativity analysis. Subtraction waveform of rare and frequent tone averages, as used for analysis of MMN, measured at Pz. Green curve: wakefulness. Red and black curves: sleep stage 2, separated according to responses without and with evoked KCs, respectively. Position of the MMN in wakefulness is indicated. Negative deflections at 100 ms during sleep may be attributed to changes in N1 amplitude rather than occurance of a MMN during sleep. (5.75 MB TIF) [file pone.0006749.s002.tif]
